# Impact of Serotonin Deficiency on Circadian Dopaminergic Rhythms

**DOI:** 10.3390/ijms25126475

**Published:** 2024-06-12

**Authors:** Giacomo Maddaloni, Noemi Barsotti, Sara Migliarini, Martina Giordano, Serena Nazzi, Marta Picchi, Francesco Errico, Alessandro Usiello, Massimo Pasqualetti

**Affiliations:** 1 Unit of Cell and Developmental Biology, Department of Biology, University of Pisa, 56127 Pisa, Italymarta.picchi@phd.unipi.it (M.P.); 2 Harvard Medical School, Department of Genetics, Harvard University, 77 Avenue Louis Pasteur, Boston, MA 02115, USA; 3 Centro per l’Integrazione della Strumentazione Scientifica dell’Università di Pisa (CISUP), 56126 Pisa, Italy; 4 CEINGE Biotecnologie Avanzate Franco Salvatore, 80131 Naples, Italy; 5 Department of Agricultural Sciences, University of Naples “Federico II”, 80055 Portici, Italy; 6 Department of Environmental, Biological and Pharmaceutical Sciences and Technologies, Università degli Studi della Campania “Luigi Vanvitelli”, 81100 Caserta, Italy; 7 Center for Neuroscience and Cognitive Systems@UniTn, Istituto Italiano di Tecnologia, 38068 Rovereto, Italy

**Keywords:** circadian rhythms, bipolar disorders, manic-like behavior, serotonin, dopamine, hyperdopaminergia, tryptophan hydroxylase 2, tyrosine hydroxylase, cholecystokinin

## Abstract

Physiology and behavior are structured temporally to anticipate daily cycles of light and dark, ensuring fitness and survival. Neuromodulatory systems in the brain—including those involving serotonin and dopamine—exhibit daily oscillations in neural activity and help shape circadian rhythms. Disrupted neuromodulation can cause circadian abnormalities that are thought to underlie several neuropsychiatric disorders, including bipolar mania and schizophrenia, for which a mechanistic understanding is still lacking. Here, we show that genetically depleting serotonin in *Tph2* knockout mice promotes manic-like behaviors and disrupts daily oscillations of the dopamine biosynthetic enzyme tyrosine hydroxylase (TH) in midbrain dopaminergic nuclei. Specifically, while TH mRNA and protein levels in the Substantia Nigra (SN) and Ventral Tegmental Area (VTA) of wild-type mice doubled between the light and dark phase, TH levels were high throughout the day in *Tph2* knockout mice, suggesting a hyperdopaminergic state. Analysis of TH expression in striatal terminal fields also showed blunted rhythms. Additionally, we found low abundance and blunted rhythmicity of the neuropeptide cholecystokinin (Cck) in the VTA of knockout mice, a neuropeptide whose downregulation has been implicated in manic-like states in both rodents and humans. Altogether, our results point to a previously unappreciated serotonergic control of circadian dopamine signaling and propose serotonergic dysfunction as an upstream mechanism underlying dopaminergic deregulation and ultimately maladaptive behaviors.

## 1. Introduction

Alternating daily cycles of light and dark produced by sunlight set the timing of biological rhythms in virtually all organisms. Animals, including humans, have evolved endogenous mechanisms, collectively known as circadian rhythms, to anticipate light/dark changes and rhythmically coordinate whole-organism behavioral and physiological responses to the natural light/dark cycle [1]. Under circadian control are fundamental processes including sleeping/waking, hormone release, metabolism, and immune responses [2]. At the neural level, circadian rhythms are orchestrated by the suprachiasmatic nucleus (SCN) of the hypothalamus, which receives direct retinal innervation and generates daily rhythms in neuronal activity that are then propagated to downstream brain regions [3,4]. Neuromodulatory systems, including serotonin (5-HT) and dopamine (DA), are under strong circadian regulation and help shape biological rhythms themselves [5,6,7,8,9,10]. Comprising the ascending arousal system [11], both neuronal populations exhibit daily oscillations in neurotransmitter gene expression as well as electrical activity, which together cause heightened release of both serotonin and dopamine during wakefulness, releasing much less during sleep [5,6,8,9,10,12]. In nocturnal animals like the laboratory mouse, peak activity and gene expression is seen during the dark phase (subjective day), while the nadir is observed during the light phase (subjective night) [5,7]. Such circadian control ensures that 5-HT- and DA-mediated behaviors and physiological processes occur at the right time of day to maximize well-being and survival. Not surprisingly, abnormalities in the circadian regulation of neuromodulatory systems have been associated with aberrant behavioral traits common in bipolar mania and schizophrenia [13]. More specifically, loss of circadian rhythmicity of DA-related genes and persistently elevated DA neuronal activity have been linked to uncontrolled behavioral euphoria and manic states in both clinical and preclinical studies, leading to the hyperdopaminergic hypothesis of bipolar mania [5,14,15,16]. Less clear is the involvement of 5-HT in manic behavior. Clinical studies have shown that both 5-HT deficit and SSRI-induced 5-HT elevation are associated with manic episodes [17], indicating a complex and still poorly understood interaction between 5-HT levels and maladaptive behavior. Filling this gap, we have recently discovered that the genetic depletion of 5-HT in Tryptophan hydroxylase 2 (*Tph2*, a rate-limiting enzyme in 5-HT synthesis) knockout (KO) mice results in hyperactivity, decreased behavioral despair, increased risk taking, and escalated aggression, reminiscent of bipolar-associated manic behavior [18]. Importantly, this behavior was normalized by the mood stabilizer valproic acid, suggesting overall high face and predictive validity [18]. Such a behavioral phenotype was associated with functional hyperactivity of a district heavily innervated by serotonergic fibers and sensitive to 5-HT levels fluctuations such as those in the hippocampus [19,20,21,22,23,24,25,26].

Here, we investigate whether the genetic depletion of 5-HT in *Tph2* KO mice impairs the circadian regulation of genes involved in DA neurotransmission in midbrain DA nuclei as a potential, additional mechanism underlying manic-like behaviors. We found that those rhythms of DA gene expression were severely blunted in *Tph2* KO mice and associated with the expression of manic-like behaviors. Our results highlight a previously unknown serotonergic control of midbrain DA neurons’ circadian rhythmicity and allow us to propose a novel framework wherein serotonergic dysfunction may be causally involved in hyperdopaminergia, ultimately contributing to manic-like behavior.

## 2. Results

**Figure 1:**
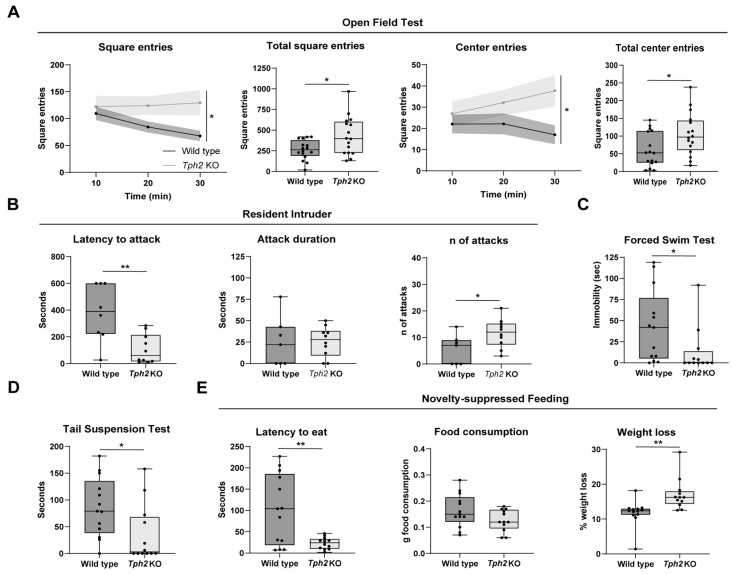
Behavioral tests on *Tph2* knockout mice. (**A**) In the graphs relative to the Open Field Test are shown the number of square entries relative to the time spent in the arena (Two-way ANOVA, interaction time × genotype, *F* (2, 30) = 3.675, *p* = 0.0374, followed by Sidak’s multiple comparison tests, at 20′, *p* = 0.0121; at 30′, *p* = 0.0001), the total number of square entries (Unpaired *t* test, t = 2.618, df = 30, *p* = 0.0137), the number of center square entries relative to the time of experiment (Two-way ANOVA, genotype effect, *F* (1, 15) = 4.677, *p* = 0.0471, followed by Sidak’s multiple comparison tests, at 30′, *p* = 0.0003), and the total number of center entries (Unpaired *t* test, t = 2.279, df = 30, *p* = 0.0300); WT n = 16; KO n = 16. (**B**) Box plot relative to the Resident–Intruder test in which are shown the latency of the first attack (Unpaired *t* test, t = 2.301, df = 15, *p* = 0.0361), the attack duration (Unpaired *t* test, t = 0.03170, df = 15, *p* = 0.9751), and the total number of attacks for WT and KO mice (Unpaired *t* test, t = 2.301, df = 15, *p* = 0.0361); WT n = 8; KO n = 10. (**C**) The graph shows the immobility of WT and KO mice in the Forced Swim Test (Unpaired *t* test, t = 2.078, df = 23, *p* = 0.0491); WT n = 13; KO n = 12. (**D**) Box plot showing the immobility measured in the Tail Suspension Test (Unpaired *t* test, t = 2.306, df = 23, *p* = 0.0305); WT n = 13; KO n = 12. (**E**) Results of the Novelty-Suppressed Feeding Test expressed as latency of eating (Unpaired *t* test, t = 3.314, df = 23, *p* = 0.0030), quantity of food consumed (Unpaired *t* test, t = 1.732, df = 23, *p* = 0.0967), and weight loss (Unpaired *t* test, t = 3.217, df = 23, *p* = 0.0038); WT n = 13; KO n = 12. Data are expressed as means ± min/max for box plots and ±s.e.m. for XY graphs, with * *p* < 0.05 and ** *p* < 0.01.

We have recently shown that *Tph2* KO mice, genetically depleted of brain 5-HT, exhibit behavioral phenotypes that are reminiscent of bipolar-associated manic behavior [18]. To confirm these findings, we subjected independent cohorts of adult *Tph2* KO mice and wild-type (WT) littermates to a battery of behavioral tests, including the Open Field Test (OFT), Resident–Intruder (RI) test, Forced Swim Test (FST), Tail Suspension Test (TST), and Novelty-Suppressed Feeding (NSF) test. The *Tph2* KO mice showed increased locomotor activity in the OFT, expressed as the number of entries into squares the arena was virtually subdivided into (Figure 1A). While the WT littermates decreased locomotion over time as they familiarized themselves with the novel environment, the KO mice showed impaired habituation (Figure 1A). Additionally, KO mice also explored the center of the arena more than the WT mice (Figure 1A). In the RI test, *Tph2* KO mice showed markedly decreased latency to attack the intruder mouse (Figure 1B) and an increased duration of single attacks (Figure 1B). When challenged in the FST (Figure 1C) and in the TST (Figure 1D), KO mice showed markedly reduced immobility compared to their control littermates. In the NSF test, KO mice exhibited strongly reduced latency to eat a food pellet placed in the center of the arena (Figure 1E) while exhibiting comparable food consumption at the end of the test (Figure 1E), even despite their greater weight loss (Figure 1E). Altogether, genetic depletion of 5-HT in *Tph2* KO mice resulted in hyperlocomotion and reduced habituation to novel environments, increased risk-taking and reduced place avoidance behavior, reduced behavioral despair, and escalated aggression—behavioral traits that have been linked to human manic states.

**Figure 2:**
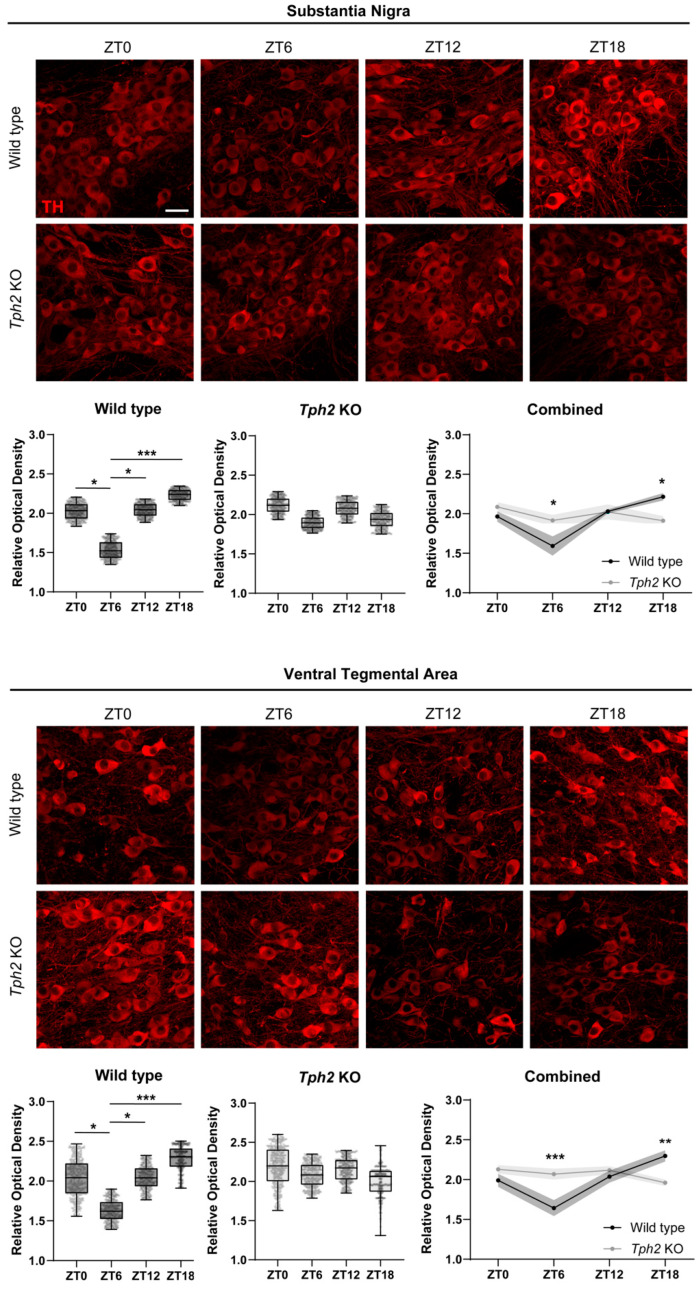
Disruption of daily oscillations of tyrosine hydroxylase in midbrain dopaminergic nuclei. Representative images showing TH immunoreactivity in both Substantia Nigra pars compacta (**top** of the panel) and Ventral Tegmental area (**bottom** panel) at ZT0, ZT6, ZT12, and ZT18 (Scale bar 30 μm). Box plots show an intra-genotype comparison of TH immunofluorescence levels measured as ROD at the different ZTs (WT cohorts in SN: One-way ANOVA, *F* (3, 12) = 14.02, *p* = 0.0003, followed by Tukey’s multiple comparison test: ZT0 vs. ZT6 *p* = 0.0124; ZT6 vs. ZT12 *p* = 0.0039; ZT6 vs. ZT18 *p* = 0.0002. KO cohorts in SN: One-way ANOVA, *F* (3, 12) = 1.523, *p* = 0.2591. WT cohorts in VTA: One-way ANOVA, *F* (3, 12) = 11.73, *p* = 0.0007, followed by Tukey’s multiple comparison test: ZT0 vs. ZT6 *p* = 0.0381, ZT6 vs. ZT12 *p* = 0.0180, ZT6 vs. ZT18 *p* = 0.0004. KO cohorts in VTA: One-way ANOVA, *F* (3, 12) = 2.431, *p* = 0.1157). TH expression levels between WT and KO mice are compared at different circadian time points. (SN: Two-way ANOVA, interaction time x genotype, *F* (3, 24) = 7.086, *p* = 0.0014, followed by Tukey’s multiple comparison test: KO vs. WT at ZT6 *p* = 0.0487, KO vs. WT at ZT18 *p* = 0.0042. VTA: Two-way ANOVA, interaction time x genotype, *F* (3, 24) = 11.24, *p* < 0.0001, followed by Tukey’s multiple comparison test: KO vs. WT at ZT6 *p* = 0.0097, KO vs. WT at ZT18 *p* = 0.0050). WT n = 5, KO n = 5 per timepoint. Data are expressed as means ± min/max for box plots and ±s.e.m. for XY graphs, with * *p* < 0.05, ** *p* < 0.01, and *** *p* < 0.001.

Loss of circadian rhythmicity in genes involved in DA neurotransmission and resulting hyperdopaminergia has been proposed to be a cellular dysfunction underlying the expression of manic-like behaviors in rodent models [5,15,16]. We next investigated whether the genetic deletion of *Tph2* impacted the circadian rhythmicity of DA-related genes in midbrain DA nuclei. We therefore generated cohorts of *Tph2* KO mice and WT littermates and perfused them for brain tissue collection at four Zeitgeber times (ZTs): ZT0, ZT6, ZT12, and ZT18, (ZT0 and ZT12 are defined as lights-on and lights-off, respectively). To analyze the circadian waveform of TH protein levels, we performed an immunofluorescence analysis followed by confocal imaging and relative optical density (ROD) measurements in single cells within the SN and VTA (Figure 2). In the WT controls, TH levels in the SN decreased during the light phase, reaching a nadir at ZT6, and then increased during the dark phase, peaking at ZT18, in keeping with previous reports (Figure 2A).

This gene expression regulation ensures that DA synthesis is higher during the subjective day (dark phase) and lower at subjective night (light phase). Strikingly, *Tph2* KO mice showed blunted circadian rhythmicity of TH protein levels in the SN, with overall high TH levels throughout the day. Specifically, we detected no difference in the mean ROD across ZTs in the KO mice, indicating impaired circadian oscillation. When compared to the WTs, *Tph2* KO mice showed higher TH levels in the SN at ZT6 but lower TH levels at ZT18 (Figure 2A). A similar scenario was observed in the VTA (Figure 2B). TH levels in the VTA of the WT mice peaked at ZT18 and reached the lowest point at ZT6. Notably, no oscillation was detected in the VTA of KO mice. Again, when compared to their WT littermates, KO mice showed higher TH levels at ZT6 but lower levels at ZT18 (Figure 2B). We next performed ROD measurements of TH levels in striatal terminal fields of SN and VTA DA neurons, namely, the Caudate/Putamen (CPu) and the Nucleus Accumbens (NAc), respectively. WT mice showed peak TH levels at ZT18 in both the CPu and NAc, in keeping with ROD measurements in the cell soma (Supplementary Figure S1). KO mice showed, however, an opposite trend: TH levels peaked at ZT6 in both the CPu and NAc (even though the difference did not reach statistical significance in the latter) and were overall lower at ZT18 (Supplementary Figure S1). To rule out potential defects in light perception that could confound interpretation, we challenged the WT and KO mice with a brief light pulse (30 min) during the dark phase (ZT16) and checked for the expression of the immediate early gene and neuronal activity marker *cFos* in the retinorecipient SCN at the end of the light pulse. Light-induced *cFos* expression highlights proper light detection from the retina and functional transmission to the central clock of the brain. The *Tph2* KO mice showed normal upregulation of *cFos* following the light pulse, suggesting functionality of the retino-hypothalamic tract, thus arguing against the notion that defects in light perception serve as an underlying mechanism (Supplementary Figure S2). Altogether, these data provide evidence for blunted oscillations in TH protein levels in the SN and VTA of *Tph2* KO mice, as well as abnormally high DA synthesis in midbrain DA neurons in addition to striatal TH levels at a time of the day where they should be at their lowest.

**Figure 3:**
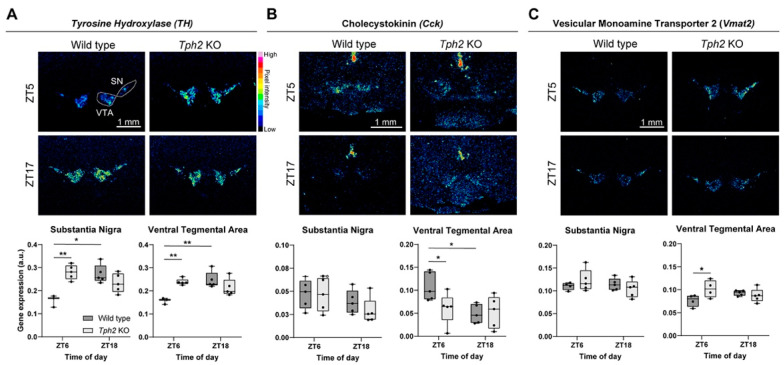
Altered daily rhythms in mRNA levels of tyrosine hydroxylase, cholecystokinin, and vesicular monoamine transporter 2. (**A**) Representative autoradiogram of coronal sections showing *Th* mRNA expression. Box plots show gene expression levels in both SN and VTA measured as relative optical density at ZT6 and ZT18 (SN: Mixed-effect model, time x genotype interaction, *F* (1, 14) = 37.47, *p* = 0.0009, followed by Sidak’s multiple comparison test: WT ZT6 vs. ZT18 *p* = 0.0016, WT vs. KO at ZT6 *p* = 0.0006. VTA: Mixed-effects model, time × genotype interaction, *F* (1, 14) = 16.92, *p* = 0.0011, followed by Sidak’s multiple comparison test: WT ZT6 vs. ZT18 *p* = 0.0008, WT vs. KO at ZT6 *p* = 0.0017). (**B**) Representative images of ^35^S ISH showing *Cck* expression and graphs of relative optical density quantification (SN: Two-way ANOVA followed by Sidak’s multiple comparison test; this analysis did not reveal any effects. VTA: Two-way ANOVA, in which no effect was found; Sidak’s multiple-comparison test: WT ZT6 vs. ZT18 *p* = 0.025, WT vs. KO at ZT6 *p* = 0.0276). (**C**) Representative images of ^35^S ISH showing *Vmat2* expression at ZT6 and ZT18. In box plots are shown the relative optical density quantifications (SN: Mixed-effecst model followed by Sidak’s multiple comparison test; no effect was found. VTA: Mixed-effect model, interaction time × genotype, *F* (1, 14) = 5.747, *p* = 0.0310, followed by Sidak’s multiple comparison test, WT vs. KO at ZT6 *p* = 0.0386). WT n = 5 KO n = 5 per timepoint. Data are expressed as means ± min/max; * *p* < 0.05 and ** *p* < 0.01. Scale bar: 1 mm.

We next analyzed whether other genes related to DA neurotransmission were deregulated in the SN and VTA of KO mice using semi-quantitative, radioactive (^35^S-based) in situ hybridization. We focused on the ZT6 timepoint, as that is when we saw the greatest difference, and compared it to the diametrically opposite ZT18 timepoint. We first checked whether the observed differences in TH protein levels were also apparent at the mRNA level. For both the SN and VTA, the results showed increased *Th* expression in KO mice compared to the WT mice at ZT6 (Figure 3A). Unlike the WT mice, no difference was detected between KO ZT6 and KO ZT18, indicating disrupted oscillation. Furthermore, *Th* expression at ZT6 in the KO mice was indistinguishable from WT ZT18, suggesting that KO mice show “subjective day-like” *Th* levels during their subjective night (Figure 3A). We next focused on the neuropeptide Cholecystokinin (Cck), a negative modulator of DA neuron activity. Cck downregulation in the VTA has been shown to be causally involved in the expression of manic-like behaviors; conversely, its upregulation has been associated with mood stabilizer treatment in both rodent models and human patients [15]. While we did not detect any change in the SN between genotypes at both time points, we observed a striking downregulation of *Cck* in the VTA of KO mice at ZT6 (Figure 3B). Moreover, while the WT mice showed higher *Cck* expression at ZT6 than ZT18, such circadian rhythm was blunted in the KO mice (Figure 3B). Analysis of other DA-related, non-circadian regulated genes, including the dopamine autoreceptor *Drd2*, the reuptake transporter *Slc6a3* (*Dat*), and the vesicular transporter *Vmat2*, revealed no differences across genotypes and time points, with the exception of increased *Vmat2* expression at ZT6 in the VTA of the KO mice (Figure 3C, Supplementary Figure S3). Taken together, these results corroborate the finding of blunted circadian rhythms in DA-related genes in the SN and VTA of *Tph2* KO mice and suggest abnormally high DA tone (the synergistic action of augmented DA synthesis (*TH*), vesicle loading (*Vmat2*), and lowered, *Cck*-mediated inhibition of DA neuron activity) in these mice at subjective night.

Finally, we investigated whether the blunted rhythms and dysregulated gene expression of DA-related genes at ZT6 stemmed from altered expression of core molecular clock genes in midbrain DA nuclei. To this end, we performed RT-qPCR for the master circadian transcription factors *Clock*, *Bmal1*, and *Rev-Erbα* (along with TH as a positive control) in midbrain explants from the WT and KO mice. While we detected robust upregulation of *TH*, as expected, no differences were found in the expression levels of these clock genes between the WT and KO mice nor in the levels of the dopamine master regulator *Nurr1* (which has been shown to compete with Rev-Erbα in regulating *Th* expression [5]) (Supplementary Figure S4). These data suggest no major molecular clock defects and further corroborate the idea of direct serotonergic control of DA neuron circadian rhythmicity.

## 3. Discussion

Behaviors, and underlying neural systems, exhibit circadian variations to ensure execution at the appropriate time of day. For example, at subjective night, neurons and molecules signaling arousal, wakefulness, and related functions are operating at their minimum levels to facilitate sleep and rest, and vice versa. Misalignment with the environmental light/dark cycle and overactivation of arousal systems (including midbrain dopamine) are thought to cause circadian and sleep defects, insomnia, hyperactivity, agitation, delusion, and illogical thinking, all hallmark symptoms of mania common to bipolar disorder and schizoaffective disorder [13,27,28]. Our study reveals in mice a previously unappreciated serotonergic control over circadian dopaminergic rhythmicity as well as an important link with the expression of behavioral outcomes that mimic human manic states.

Persistently elevated DA signaling, a core neurochemical dysfunction underlying manic symptoms, is supported by both preclinical studies and clinical observations [28]. Such DA neurochemical imbalance is thought to arise from a loss of daily DA oscillations and consequently high levels of DA throughout the day, as seen in several knockout mouse models for clock genes [5,15,16]. Blunted circadian rhythms in DA-related gene expression and DA neuronal activity have been described in mouse mutants for the *Clock* gene and for the *Rev-Erbα* gene, thus suggesting a strong influence of the molecular clock on DA rhythmicity [5,16,29]. Here, we provide evidence of a parallel pathway controlling daily DA level oscillations, seemingly independent from the molecular clock control, that involves the neuromodulator 5-HT. *Tph2* KO mice, genetically depleted of brain 5-HT, showed blunted circadian rhythms in TH protein and mRNA levels in midbrain DA nuclei as well as in striatal terminal fields (Figure 2, Supplementary Figure S1). As TH levels were high across all the time points analyzed, this loss of rhythmicity resulted most likely in persistently high DA tone throughout the day. This was most evident during the light phase (subjective night for the mice), when mice normally spend around 70% of their time sleeping, and reasonably caused the observed hyperactive, manic-like behavior. Interestingly, *Tph2* deletion in adult mice also caused hyperactive behavior [30], suggesting that the phenotype we observed was not generated by secondary rearrangements caused by 5-HT loss during brain development and circuits maturation.

In addition to impaired TH rhythms, we also found a blunted rhythm and lower levels of the neuropeptide *Cck* in the VTA of KO mice (Figure 3B). Interestingly, a similar phenotype has been observed in *Clock* mutant mice, and it was causally linked to manic-like behaviors [15]. As Cck has been shown to negatively modulate the activity of DA neurons, lower levels seen in the VTA of KO mice might synergize with higher TH levels, therefore boosting DA neuron activity and DA release. We also found upregulated expression of the monoamine transporter *Vmat2* in the middle of the light phase (Figure 3C), further supporting the hypothesis of increased DA release.

Our experiments suggest that loss of DA rhythmicity is not due to defects in light perception (and therefore the internal encoding of external light conditions) or a disrupted molecular clock. *Tph2* KO mice showed normal induction of the immediate early gene *cFos* in the SCN following a light pulse in the middle of the dark phase, indicating intact communication from the retina to the brain (Supplementary Figure S2). When analyzed at ZT6, the time point that showed the most differences, the expression levels of core clock genes (*Clock*, *Bmal1*, *Rev-Erbα*) in the midbrain were comparable between the KO and WT mice (Supplementary Figure S4). How does 5-HT depletion impact the rhythmicity of DA-related genes? While we do not provide an answer for this important question, we speculate that loss of 5-HT, and consequently loss of a rhythmic signal itself onto DA neurons, leaves them “uninformed” about the external light/dark conditions. The notion that serotonin and dopamine might have mutually opposing functions in mood regulation is already established [31,32]. Our work elaborates this further and suggests that the serotonergic pathway might be the primary brain pathway that provides light/dark information to midbrain DA neurons, therefore dictating daily rhythms in gene expression and most likely in firing activity. Future studies are needed to answer these outstanding questions.

Finally, it is worth noting that genetic 5-HT depletion results in both disrupted TH circadian rhythmicity, as described here, as well as functional hyperactivity in the hippocampus, as shown previously in our lab [18], which may synergize or underlie specific, non-overlapping aspects of the observed manic-like behaviors. Regardless, this highlights an important, global form of serotonergic control over the functionality of brain structures critically implicated in the modulation of emotional behavior and suggests that serotonergic dysfunction might be an upstream mechanism underlying manic-related neuropsychiatric disorders.

## 4. Materials and Methods

### 4.1. Animals

*Tph2* KO mice [22] and control WT littermates were housed in standard Plexiglas cages under a constant temperature/humidity (22 ± 1 °C, 50–60%) and maintained on a 12/12 h light/dark cycle, with food and water available ad libitum. All animals used in each experiment had a C57BL/6 genetic background. Experimental protocols were conducted in accordance with the Ethical Committee of the University of Pisa and approved by the Veterinary Department of the Italian Ministry of Health.

### 4.2. Behavior

All behavioral tests were performed on independent cohorts for each test in accordance with standard protocols during the light phase of the light/dark cycle (11:00 am–13:00 pm). Depression-like behaviors were assessed using the Forced Swim Test (FST) and the Tail Suspension Test (TST) (WT n = 13; KO n = 12). In the FST, animals were placed in a 5L Plexiglass beaker containing 4L of 26 °C water and a video-recorder for 6 min. In the TST, mice were hung by their tails from a bar placed 50 cm from the ground with a piece of autoclave tape and recorded in a 6 min session. In both tests, minutes 2 to 6 were analyzed for immobility time, which was intended to be the absence of any active movement of the paws.

Anxiety-like behaviors were analyzed by applying Novelty-Suppressed Feeding (NSF) test and Open Field Test (OFT). In the NSF test, food was removed from the mice’s cages 24 h before testing. The next day, mice were placed for 10 min in a bright-white arena (38 × 35 × 20 cm) without bedding and with a food pellet at the center. Mice were video-recorded, and latency of feeding was assessed offline. After the test, to avoid generating confounding effects of feeding behavior on anxiety, hunger was measured by weighing a single pellet of food placed in the home cage before and after 5 min (WT n = 13; KO n = 12). The OFT was performed in a white arena (38 × 35 × 20 cm); mice were placed at the center of the arena and allowed to freely explore the chamber. Activity of the mice was video-recorded, and analyses were performed by subdividing the arena into peripheral and central squares. Number of entries in the squares in 30 min time window, number of entries in the central squares, and total square entries were quantified (WT n = 16; KO n = 16).

Aggressive behavior was assessed in a Resident–Intruder (RI) test. Briefly, one male (resident) of both genotypes was housed with a female one week before the test. Then, the female was removed from the cage, an unfamiliar male (intruder) was introduced into the cage, and animals were video-recorded for 10 min. Lateral threats and clinch attacks were considered signs of aggression. Latency of the first attack, attack duration, and number of attacks were measured (WT n = 8; KO n = 10).

### 4.3. Immunohistochemistry

Animals of both genotypes were sacrificed at ZT0, ZT6, ZT12, and ZT18 (n = 5 each time point and genotype). Mice were transcardially perfused with PFA 4%, and brains were removed, dissected, and post-fixed overnight (O/N) in PFA 4% at 4 °C. Next day, brains were cut into 50 µm coronal sections with a vibratome (Leica). Immunohistochemistry was performed on free-floating sections, in parallel for both genotypes and all ZTs. Briefly, sections were incubated in blocking solution (PBS 1×, Triton X-100 0.5% and Horse serum 5%) for 1h at room temperature (RT). Next, samples were incubated in primary antibody solution (Mouse anti Tyrosine Hydroxylase 1:800 Millipore MAB318, Burlington, MA, USA) O/N at 4 °C. Next day, sections were rinsed in washing solution (PBS + Triton X-100 0.5%) three times. Tissue was incubated in the secondary antibody solution (Goat anti Mouse Rhodamine Red-X, 1:500, ThermoFisher Scientific R6393, Waltham, MA, USA). On the next day, three washes were performed using the washing solution. Immunohistochemistry without primary antibody was performed in parallel to obtain background signal. Sections were mounted using AquaPolymount (Polysciences, Warrington, PA, USA).

Images of TH immunopositive cells in Substantia Nigra and Ventral Tegmental Area were acquired using a Nikon A1 confocal microscope equipped with 60× Plan-Apo objective. Z series of 41 stacks were acquired at 1024 × 1024 pixel resolution (pixel size: 0.21 µm), with a z-step of 0.125 µm for a total thickness of 5 µm. Confocal images of Caudate/Putamen (CPu) and Nucleus Accumbens (NAc) were imaged using a 10× plan-apochromat objective. Images were acquired at 1024 × 1024 pixel resolution (pixel size: 1.24 µm), with a z-step of 1 µm for a total thickness of 5 µm.

### 4.4. Fluorescence Intensity Analysis

TH expression levels in SN and VTA were analyzed by measuring fluorescence signal in single cells (400 cells each animal, n = 5 per timepoint), excluding the nucleus, using ImageJ software. Mean fluorescence signal was normalized on background signal calculated on control sections stained without primary antibody.

ImageJ software was used to measure TH expression in CPu and NAc. Fluorescence signal was analyzed for two images acquired in three subsequent brain sections relative to CPu and NAc for each animal (n = 5 per timepoint). Background signal was calculated on sections immunostained without primary antibody.

### 4.5. In Situ Hybridization

Digoxigenin (DIG) (*cFos* 1.8 Kb), or ^35^S-labeled (*TH*, 1.5 Kb; *Cck*, 0.4 Kb; *Slc6a3*, 0.7 Kb; *Vmat2*, 1.0 Kb; *Drd2*, 1.0 Kb) antisense riboprobes, were used. Probes used are as follows: *Cck*, nucleotides 68–452 (NM_001284508.3); *Slc6a3*, nucleotides 170–855 (NM_010020); *Vmat2*, nucleotides 166–1149 (NM_172523.3); *cFos* [33,34,35].

For radioactive in situ hybridization analyses, animals (n = 5 each genotype) were sacrificed at ZT6 and ZT18, and fresh brain tissue was embedded in Tissue Tek (Sakura) and frozen at −80 °C. Brains were then cryo-sectioned in the coronal plane (14 µm thick). In situ hybridization was performed as previously described using ^35^S-labeled antisense RNA probe. Sections were exposed to Biomax MR X-ray films (Kodak) [36]. For each probe, sections from different genotypes were processed in parallel to minimize experimental variability. Images were acquired with a MacroFluo microscope (Leica) equipped with Nikon NIS-Elements software and processed with ImageJ software to convert them into 8-bit images for signal quantification.

For *cFos* expression analysis, animals were exposed to a 30 min light pulse 3 h after the beginning of the dark phase. At the end of the light pulse, animals were sacrificed, and their brains were immediately dissected and frozen in OCT. Control animals were not exposed to light pulse, and they were sacrificed during the dark phase. ISH analysis was performed according to protocols previously described [37], using digoxigenin-labeled antisense *cFos*-RNA probe. Staining was performed using NBT/BCIP (Roche) as substrate for alkaline phosphatase.

### 4.6. Quantitative Reverse Transcriptase-PCR

Midbrain tissue punches were harvested from WT and *Tph2* KO mice at ZT6 (WT n = 4; KO n = 4) for quantitative reverse transcriptase PCR (RT-qPCR) analysis. RNA was extracted using TRIzol reagent (Life Technologies, Carlsbad, CA, USA), following the manufacturer’s instructions. The cDNA was synthesized by using 500 ng of RNA and the GoScript Reverse Transcription System (Promega, Madison, WI, USA) with Random Primers and processed under the following cycling conditions: 70 °C 5 min, 25 °C 5 min, 42 °C for 60 min, 70 °C for 15 min, and holding at 4 °C. The synthesized cDNA was combined with GoTaq(R) qPCR Master Mix (Promega) on a QuantStudio3 PCR system (Applied Biosystems, Waltham, MA, USA) and specific primers (Supplementary Table S1). PCR conditions included an initial denaturation step at 95 °C for 2 min, followed by 40 cycles at 95 °C for 15 s, 60 °C for 1 min, and 95 °C for 15 s. mRNA quantifications were normalized to the housekeeping gene β-actin, and each reaction was induced in three replicates. Relative expression changes between conditions were calculated using the 2^−ΔΔCT^ method.

### 4.7. Statistical Analysis

Statistical analyses were carried out using GraphPad Prism 9.0. Behavioral tests were analyzed through unpaired *t* test or Two-way ANOVA followed by Sidak’s correction for multiple comparisons. Results of intra-genotype comparison of TH levels and RT-qPCR results were tested by applying One-way ANOVA, while differences in TH fluorescence signal between WT and KO mice at different timepoints and ISH results were statistically validated using two-way ANOVA or a mixed-effects model, followed by conducting Sidak’s or Tukey’s correction for multiple comparisons. RT-qPCR data were analyzed through multiple t-test comparisons. Not significant: *p* > 0.05; * *p* < 0.05; **p < 0.01; *** *p* < 0.001.

## Data Availability

The original contributions presented in the study are included in the article/supplementary material; further inquiries can be directed to the corresponding authors.

## Supplementary Materials

The following supporting information can be downloaded at https://www.mdpi.com/article/10.3390/ijms25126475/s1. Figure S1: Blunted circadian rhythms of tyrosine hydroxylase in dopaminergic target regions; Figure S2: Assessment of suprachiasmatic nuclei response to light exposure; Figure S3: Analysis of mRNA levels of dopamine receptor type 2 and Dopamine transporter in dopaminergic nuclei; Figure S4: Analysis of clock-related genes in dopaminergic nuclei; Table S1: Oligo DNA sequence for qPCR primers.
