# Supplementary material for: Impact of Serotonin Deficiency on Circadian Dopaminergic Rhythms
Source: Int J Mol Sci. 2024 Jun 12;25(12):6475. doi: 10.3390/ijms25126475 (PMC11203511; doi:10.3390/ijms25126475)
Supplement: Supplementary file 1 [file ijms-25-06475-s001.zip › ijms-3034903-supplementary.pdf]

Serotonergic control of circadian dopamine rhythmicity

Giacomo Maddaloni [1,2,*,§], Noemi Barsotti [1,3,§], Sara Migliarini [1], Martina Giordano [1], Serena Nazzi [1], Marta Picchi [1], Francesco Errico [4], Alessandro Usiello [4,5] and Massimo Pasqualetti [1,3,6,*]

[1] Department of Biology, Unit of Cell and Developmental Biology, University of Pisa, Pisa 56127, Italy
[2] Present Address: Harvard Medical School, Department of Genetics, Harvard University, 77 Avenue Louis Pasteur, Boston, MA 02115, USA
[3] Centro per l'Integrazione della Strumentazione Scientifica dell'Università di Pisa (CISUP), Pisa 56126, Italy
[4] CEINGE Biotecnologie Avanzate Franco Salvatore, Naples, Italy
[5] Department of Agricultural Sciences, University of Naples "Federico II", 80055 Portici, Italy
[6] Department of Environmental, Biological and Pharmaceutical Sciences and Technologies, Università degli Studi della Campania "Luigi Vanvitelli", 81100 Caserta, Italy
[7] Center for Neuroscience and Cognitive Systems@UniTn, Istituto Italiano di Tecnologia, Rovereto 38068, Italy
* Correspondence: G.M Giacomo_Maddaloni@hms.harvard.edu; M.P. massimo.pasqualetti@unipi.it

§ Equal contribution

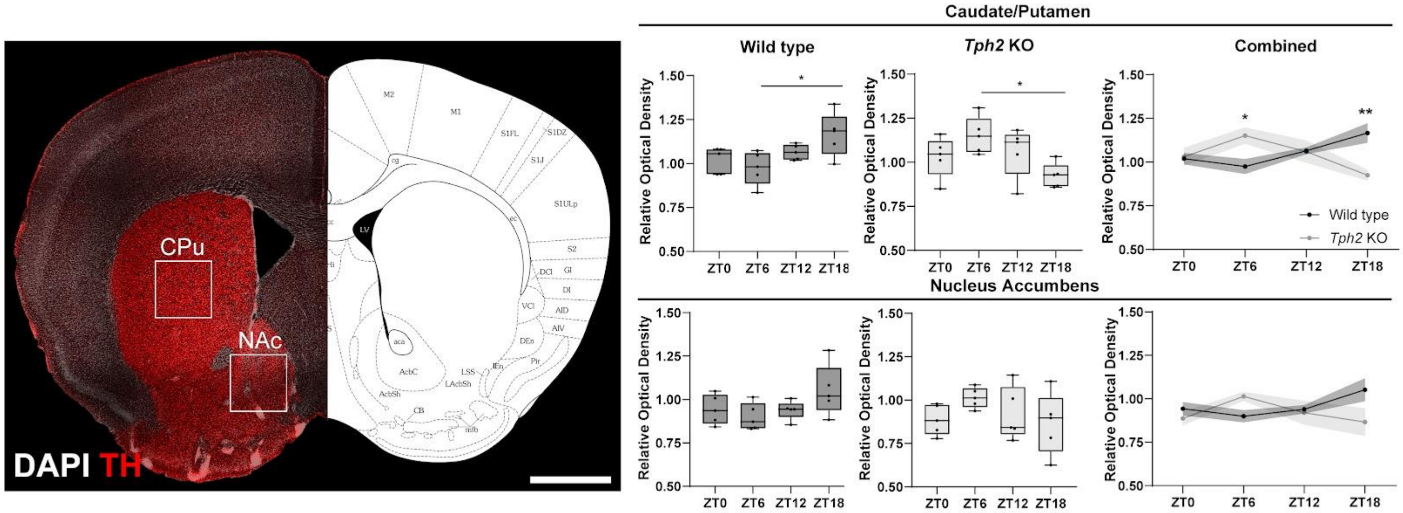

Supplementary Figure S1: Blunted circadian rhythms of tyrosine hydroxylase in dopaminergic target regions. Large image showing the analyzed region in CPu and NAc (Scale bar:1mm). Intra-genotype comparison of TH expression level measured at ZT0, ZT6, ZT12 and ZT18. (WT cohorts in CPu: One-way ANOVA, F(3, 16)=4.197, p=0.0227, followed by Tukey's multiple comparisons test: ZT6 vs ZT18 p=0.0179. KO cohorts in CPu: One-way ANOVA, F(3, 16)=3.563, p=0.0390, followed by Tukey's multiple comparisons test, ZT6 vs ZT18 p=0.0238. WT cohorts and KO in NAc: One-way ANOVA, no significant effect found). TH expression levels are compared between WT and KO at the different circadian timepoint (CPu: Two-way ANOVA, interaction time x genotype, F(3, 32)=7.306, followed by Sidak's multiple comparisons test, WT vs KO at ZT6 p=0.0362, at ZT18 p=0.0025. NAc: Two-way ANOVA, interaction time x genotype, F(3, 32)=2.906, p=0.0497, followed by Sidak's multiple comparisons test, no significant difference found). WT n=5, KO n=5 per timepoint. Data expressed as mean ± min/max for box plots, ± s.e.m. for XY graphs, *p < 0.05, **p < 0.01, ***p < 0.001.

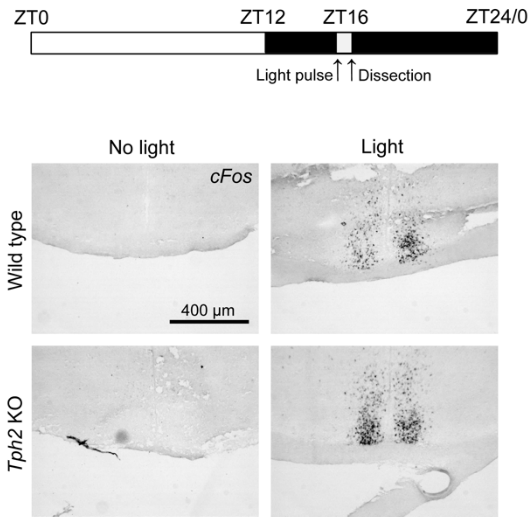

Supplementary Figure S 2: Assessment of suprachiasmatic nuclei response to light exposure. Schematic representation of the experimental paradigm and representative coronal section of DIG-labeled ISH for *cFos*. Induction of the immediate early gene *cFos* in the suprachiasmatic nucleus of the hypothalamus of Tph2 KO mice reveals that serotonin depletion does not affect light perception. Scale bar 400 µm.

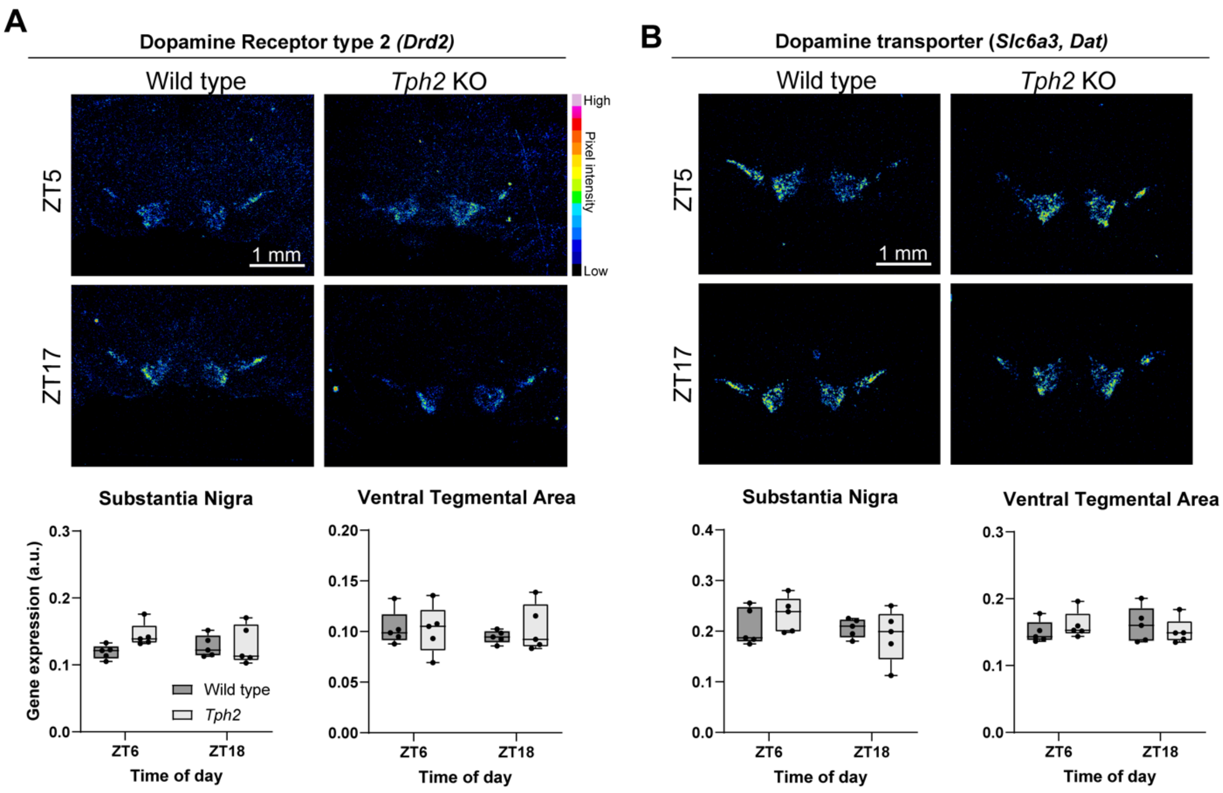

Supplementary Figure S3: Analysis of mRNA levels of Dopamine Receptor type 2 and Dopamine transporter in dopaminergic nuclei. A) Representative autoradiogram of coronal sections showing *Drd2* mRNA expression. Box plots are showing gene expression levels in both SN and VTA measured as relative optical density at ZT6 and ZT18 (SN: two-way ANOVA followed by Sidak's multiple comparisons test, no significant effect found. VTA: two-way ANOVA followed by Sidak's multiple comparisons test, no significant effect found). **B**) Representative images of ^{35}S ISH showing *Dat* expression and graphs of relative optical density quantification (SN: two-way ANOVA followed by Sidak's multiple comparisons test, no significant effect found. VTA: two-way ANOVA followed by Sidak's multiple comparisons test, no significant effect found). Scale bar 1 mm

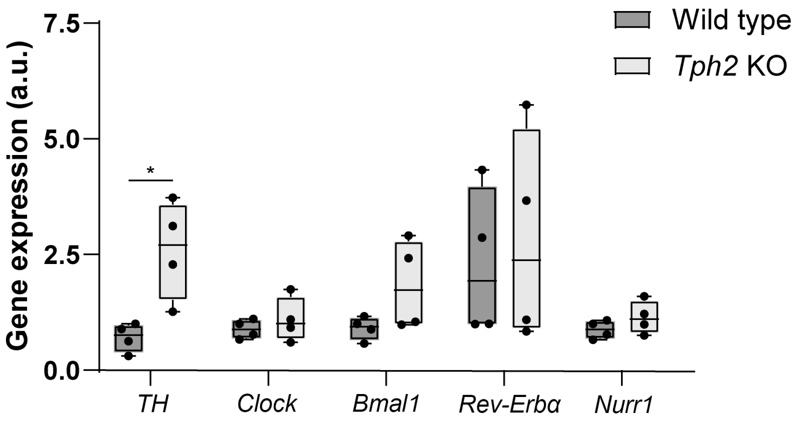

Supplementary Figure S4: Analysis of Clock-related genes in dopaminergic nuclei. RT-qPCR showing *TH*, *Clock*, *Bmal1*, *Rev-Erbα*, and *Nurr1* expression level at ZT6 in midbrain (multiple *t* tests corrected for multiple comparisons: *TH*: *t*=3.401, *df*=6, p=0.0144; *Clock*: *t*=0.7793, *df*=6, p=0.4654; *Bmal1*: *t*=1.859, *df*=6, p=0.1123; *Rev-Erbα*: *t*=0.3796, *df*=6, p=0.7173; *Nurr1*: *t*=1.282, *df*=6, p=0.2472). WT n=4 KO n=4. Data expressed as mean ± min/max., $^*p < 0.05$, $^{**}p < 0.01$, $^{***}p < 0.001$.

Gene	Forward	Reverse
β-actin	5′-AGGCATCACTATTGGCAACGA-3′	5′-CCGATCCACACAGAGTACTTG-3′
TH	5′-AAGATCAAACCTACCAGCCG-3′	5′-TACGGGTCAAACTTCACAGAG-3′
Clock	5′-TCTCAAGGAAGCACTGGAAAG-3′	5′-CAGTAGGGATCTTTGTCGGTG-3′
Bmal1	5′-ACCAACCCATACACAGAAGC-3′	5′-GACAGACTCGGAGACAAAGAG-3′
Rev-Erbα	5′-GACCTTTCTCAGCACGACC-3′	5′-CATCACTGTCTGGTCCTTCAC-3′
Nurr1	5′-TGTTCGCACTTGTGAGGG-3′	5′-AGCTAGGCACTTCTGAAACC-3′

Table S1: Oligo DNA sequence for RT-qPCR primers.
